# Progression and metastasis of small cell lung carcinoma: the role of the PI3K/Akt/mTOR pathway and metabolic alterations

**DOI:** 10.1007/s10555-021-10012-4

**Published:** 2021-12-27

**Authors:** Ildiko Krencz, Daniel Sztankovics, Titanilla Danko, Anna Sebestyen, Andras Khoor

**Affiliations:** 1 1st Department of Pathology and Experimental Cancer Research, Semmelweis University, Ulloi ut 26., H-1085 Budapest, Hungary; 2 Department of Laboratory Medicine and Pathology, Mayo Clinic, 4500 San Pablo Road, Jacksonville, FL 32224 USA

**Keywords:** Small cell lung carcinoma, Metastasis, mTOR, Metabolism

## Abstract

Small cell lung carcinoma (SCLC) is characterized by high metastatic rate and poor prognosis. The platinum-based chemotherapy still represents the backbone of the therapy; however, acquired resistance develops almost in all patients. Although SCLC has been formerly considered a homogeneous disease, recent advances in SCLC research have highlighted the importance of inter- and intratumoral heterogeneity and have resulted in the subclassification of SCLC. The newly described SCLC subtypes are characterized by distinct biological behavior and vulnerabilities that can be therapeutically exploited. The PI3K/Akt/mTOR pathway is frequently affected in SCLC, and its activation represents a promising therapeutic target. Since the mTOR pathway is a master regulator of cellular metabolism, its alterations may also influence the bioenergetic processes of SCLC cells. Despite the encouraging preclinical results, both mTOR and metabolic inhibitors have met limited clinical success so far. Patient selection for personalized therapy, the development of rational drug combinations, and a better understanding of heterogeneity and spatiotemporal evolution of the tumor cells may improve efficacy and can help to overcome acquired resistance. Here we provide a summary of current investigations regarding the role of the mTOR pathway and metabolic alterations in the progression and metastasis formation of SCLC.

Small cell lung carcinoma (SCLC) represents 15% of lung cancers, which are the leading cause of cancer death worldwide [1]. SCLC is almost always caused by smoking [2] and is characterized by a high proliferation rate, early metastases, and an exceptionally poor prognosis [1]. Despite excellent initial response to platinum-based conventional chemotherapy, acquired resistance and disease progression occur frequently [3]. A high mutational rate and genomic instability likely contribute to the unrelenting behavior of SCLC [4].

Concomitant inactivation of the *TP53* and *RB1* genes, encoding for the tumor suppressors p53 and RB, is commonly observed in SCLC cases [5]. Other genetic changes, such as *MYC* amplification or mutations of genes coding for proteins involved in cell cycle regulation, apoptosis, chromatin remodeling, or the mechanistic target of rapamycin (mTOR) signaling pathway, can also contribute to tumorigenesis and progression; however, the genetic landscape does not seem to determine SCLC subtypes [4, 5]. Recently, four major subtypes have been identified based on the expression of the transcription factors achaete-scute homologue 1 (*ASCL1*, SCLC-A subtype), neurogenic differentiation factor 1 (*NEUROD1*, SCLC-N subtype), POU class 2 homeobox 3 (*POU2F3*, SCLC-P subtype), and yes-associated protein 1 (*YAP1*, SCLC-Y subtype) [6]. SCLC-A and SCLC-N subtypes are associated with high expression of neuroendocrine (NE) markers, whereas SCLC-P and SCLC-Y subtypes have been reported as so-called “NE-low” SCLCs [6, 7].

Despite its relatively uniform histological appearance, the importance of biological heterogeneity of SCLC has started to emerge in the past few years [7]. Besides the intertumoral heterogeneity observed in molecular alterations and expression of the above-mentioned transcription factors, significant intratumoral heterogeneity has also been described suggesting that the coexistence of cancer cells with distinct vulnerabilities and resistance mechanisms within the tumor mass can help the evolvement of chemoresistance, disease progression, and metastasis formation [8, 9].

Among all solid tumors, SCLCs have one of the highest metastatic potential. Most patients have metastases at the time of the diagnosis, frequently involving the lymph nodes, brain, liver, and bones [10]. The high metastatic tendency is underlined by the exceptionally high number of the circulating tumor cells (CTCs) in SCLC patients, providing a unique opportunity for metastatic spread [11]. Given metastasis formation is a major cause of morbidity and mortality of SCLC patients, targeting metastases is critical for the development of new treatment strategies.

The standard platinum-etoposide combination still represents the backbone of therapy. However, after a gap of many years, a few new drugs have been approved for the treatment of SCLC: the alkylating agent lurbinectedin and the immune checkpoint inhibitors nivolumab and pembrolizumab have been granted accelerated approval for second- and third-line treatment [12–14]. Additionally, the durvalumab plus platinum-etoposide combination has been approved as first-line treatment for extensive-stage SCLC [15], offering new hope for a subgroup of the patients. Although these results are encouraging, a better understanding of the disease is needed to uncover novel vulnerabilities that can serve as therapeutic targets.

## mTOR pathway alterations in small cell lung carcinomas

**Fig. 1:**
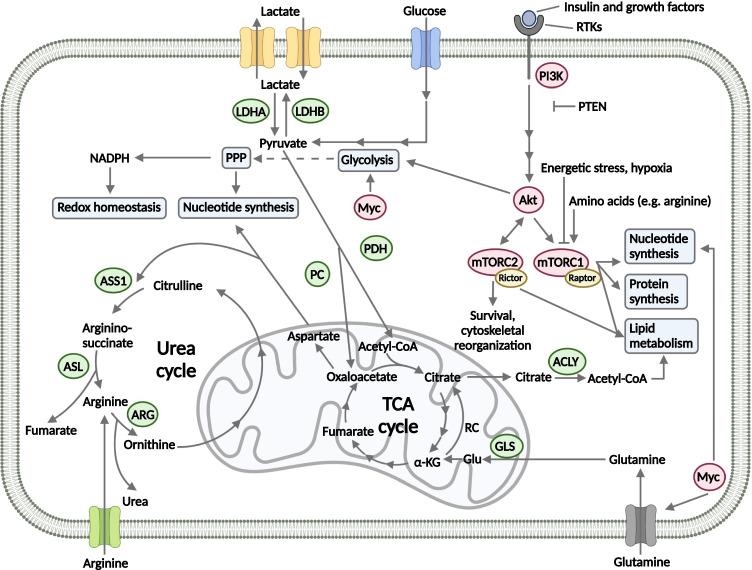
Interplay between signaling and metabolic pathways in small cell lung carcinoma. The PI3K/Akt/mTOR pathway is frequently affected in SCLC. The mTOR kinase forms the catalytic subunit of two distinct multiprotein complexes, mTORC1 and mTORC2. The mTOR complexes regulate metabolic processes: mTORC1 promotes protein and nucleotide synthesis, whereas lipid metabolism can be influenced by both mTORC1 and mTORC2 [16]. Besides the mTOR pathway, *MYC* alterations can also affect bioenergetic processes, such as glycolysis, nucleotide synthesis, and glutamine metabolism [26]. In contrast, metabolic changes can influence the activity of signaling pathways. mTORC1 is inhibited under energetic stress or hypoxia; additionally, its activity is regulated by the availability of certain amino acids, such as leucine and arginine [27]

The mTOR protein is a serine/threonine kinase that forms the catalytic subunit of two multiprotein complexes, namely mTOR complex 1 (mTORC1) and mTOR complex 2 (mTORC2) (Fig. 1). The mTOR complexes differ not only in protein subunits but also in their functions and inhibitor sensitivity. mTORC1 mainly regulates cell growth and proliferation, whereas mTORC2 has a role in cytoskeletal reorganization and regulation of cell death; additionally, it seems to be less sensitive to rapamycin analogs (so-called rapalogs) as compared to mTORC1 [16]. The mTOR complexes are an integral part of the phosphatidylinositol 3‑kinase (PI3K)/protein kinase B (Akt)/mTOR pathway and regulate several essential cell functions in response to diverse environmental and nutritional signals, such as adenosine triphosphate (ATP), oxygen, and amino acid levels in the microenvironment [16, 17]. In addition to its fundamental functions in energy and nutrient sensing, the mTOR pathway is considered as a master regulator of cellular metabolism as it orchestrates glucose, amino acid, nucleotide, fatty acid, and lipid metabolism [18]. Both mTOR complexes can promote the Warburg effect: mTORC1 regulates glucose metabolism mainly through the transcription factors HIF1α and MYC, whereas mTORC2 can also increase the expression of glycolytic enzymes [19, 20]. The role of mTOR complexes in the activation of other bioenergetic processes (glutaminolysis, pentose phosphate pathway (PPP), lipid synthesis, and fatty acid β-oxidation) has also been described, mainly via the regulation of the expression of different transcription factors (e.g., MYC, SREBP) as well as the activation or inhibition of metabolic enzymes (e.g., glucose transporters, hexokinase, glucose-6 phosphate dehydrogenase, glutamine synthetase, and fatty acid synthase) (Fig. 1) [18, 21–25].

Hyperactivation of the mTOR pathway represents a promising candidate for the personalized therapy of SCLC. Recent advantages in molecular profiling have revealed that genetic alterations of certain genes encoding for proteins of the mTOR pathway frequently occur in SCLCs [28–30]. Based on a comprehensive genomic analysis, genetic alterations of the PI3K/Akt/mTOR pathway (including *PIK3CA*, *PTEN*, *AKT2*, *AKT3*, *RICTOR*, and *MTOR* genes) are present in 36% of the SCLC patients [30]. Among these genes, *RICTOR* amplification, coding for the scaffold protein of mTORC2, has been observed as the most common targetable genetic alteration in SCLC with a frequency of 6 to 15% in different studies (Table 1) [31–33]. The expression of mTOR pathway activation markers has also been studied by immunohistochemistry, and it has been described that the expression of p-mTOR, a marker for mTOR kinase activity, is present in more than half (55 to 87.8%) of the cases [34, 35]. Additionally, mTOR kinase has been recently identified as an essential kinase of SCLC in a study including *in vitro* and *in vivo* models [36]. Expression of Rictor, the characteristic scaffold protein of mTORC2, has been detected in 37% of the patients, whereas positive staining for p-p70S6K (a downstream target of mTORC1) and p-Akt (a downstream kinase of mTORC2) has been found in 84% and 42% of the cases, respectively [31, 35].

**Table 1:** Genetic alterations of the PI3K/Akt/mTOR pathway and protein expression of mTOR activity markers in small cell lung carcinoma

Gene	Protein	Alteration type	Frequency (%)	References
*PIK3CA*	PI3K p110α	Mutation	3–6	[28–30]
		CNG	2	
*PTEN*	Pten	Mutation	2–6	[28, 29]
*AKT1/AKT2/AKT3*	Akt1/Akt2/Akt3	Mutation	2	[28–31]
		CNG	< 1	
		p-Akt expression (Ser473)	42	
*MTOR*	mTOR	Mutation	2–8	[28–30, 34, 35]
		CNG	< 1	
		p-mTOR expression	55–87.8	
*RICTOR*	Rictor	Mutation	2–3	[28–31, 33, 37]
		CNG	6–15	
		Rictor expression	37	
*RPS6KB1*	p70S6K	Mutation	0–3	[5, 35]
		CNG	0	
		p-p70S6K expression	84	

## Metabolic characteristics of small cell lung carcinoma

Tumor initiation and progression require metabolic reprogramming that is now considered as a hallmark of cancer [38]. Increasing evidence suggests that bioenergetic processes also have an important role in the pathobiology of SCLC, and metabolic liabilities can be therapeutically exploited. Most of the SCLC cases are characterized by elevated glucose uptake that can be detected by ^18^F-fluorodeoxyglucose positron emission tomography/computed tomography (FDG-PET/CT). Glucose fuels different metabolic pathways such as glycolysis, tricarboxylic acid (TCA) cycle, nucleotide synthesis via the PPP, and amino acid biosynthesis [39]. As both maximum and integrated standardized uptake value, metabolic tumor volume, and total lesion glycolysis have been described as negative prognostic factors in SCLC, FDG-PET/CT can be useful in patient management and treatment stratification, especially in limited disease [40–43]. In line with these observations, a retrospective study analyzing 98 SCLC patients has found an association among elevated serum lactate dehydrogenase (LDH) level, the enzyme that catalyzes the final step of glycolysis, decreased overall and progression-free survival [43]. However, higher serum LDH levels and glucose transporter 1 (GluT-1) expression have correlated with better objective response rate in SCLC patients who received chemoradiation [44].

Although SCLCs are thought to be highly glycolytic, other bioenergetic alterations, such as lipid, nucleotide, and amino acid metabolism, may also support the rapid proliferation of tumor cells and disease progression. A metabolomics study has found higher expressions of carnitine palmitoyltransferase 1A and 2, key enzymes of fatty acid β-oxidation, in a single SCLC cell line (NCI-H446) as compared to non-small cell lung cancer (A549) and control epithelial cells (BEAS-2B) [45]. However, cell lines of similar lineages can be metabolically diverse [46], and, currently, there are no additional studies available to support the above observation. In addition, mevalonate pathway and cholesterol metabolism also seem to be deregulated in a subset of the cases [47], and higher serum low-density lipoprotein level and low-density lipoprotein receptor expression of the tumor cells are related to unfavorable clinical outcome [48].

An adequate and balanced supply of nucleotides is necessary for accurate DNA replication and repair and can be provided by the PPP as well as salvage and *de novo* pathways of nucleotide synthesis [49]. To fulfill the nucleotide demands of highly proliferative SCLC cells, tumor cells upregulate nucleotide biosynthetic pathways. It has been described that ribonucleotide reductase (RNR), a rate-limiting enzyme of *de novo* deoxyribonucleotide triphosphate synthesis, can serve as a therapeutic target in SCLC as its large subunit (RRM1) induces DNA damage response and decreases the number of cells with S phase cell cycle arrest as well as it is required for the growth of SCLC cells [50]. Inosine-5′-monophosphate dehydrogenase (IMPDH) enzymes, which are involved in purine biosynthetic processes, have also been found to be elevated in a subset of SCLC cases [51]. Glucose serves as one of the major sources of nucleotide synthesis via the PPP; however, amino acids, such as glutamine, also contribute to the supply of not only carbon but also nitrogen, the latter of which is also required for the biosynthesis of pyrimidine and purine nucleotides [52, 53].

Besides their roles in energy generation, nucleotide biosynthesis, and maintenance of cellular redox homeostasis, amino acids play a complex role in the metabolic regulation of SCLC. Accumulating evidence supports that SCLC cells are auxotrophic for arginine meaning that tumor cells are unable to synthesize it and are dependent on extracellular arginine because of the lack or low expression of argininosuccinate synthetase 1 (ASS1) [54, 55]. ASS1 is required for arginine synthesis in the urea cycle (see Fig. 1), and its low expression has also been described as a possible biomarker for sensitivity to arginine deprivation therapy. An immunohistochemical analysis of 16 cases has found frequent loss of ASS1 in SCLC: 45% of the cases have demonstrated faint or no ASS1 expression. Additionally, PEGylated arginine deiminase (ADI-PEG20), a drug that lowers arginine level, caused dose-dependent antiproliferative effect in ASS-deficient SCLC cell lines [56]. Proline and glutamine are precursors of arginine synthesis and exert different functions in tumor growth and progression, as they also contribute to the synthesis of nucleotides and other amino acids as well as the maintaining of redox homeostasis [57].

Cancer stem cells (CSCs) are major determinants of intratumoral heterogeneity, treatment resistance, and recurrence of SCLC [58]. Recently, an *in vitro* study focusing on the metabolism of CSCs in an SCLC cell line has revealed that putative CSCs (urokinase-type plasminogen activator receptor positive SCLC cells) prefer oxidative phosphorylation over glycolysis to meet their bioenergetic requirements, have lower oxygen consumption and lower extracellular acidification rates than non-stem cancer cells, and are more sensitive to the inhibition of oxidative phosphorylation [59]. CSCs are believed to be more resistant to radiochemotherapy than the non-stem cancer cells, and, therefore, targeting the metabolism of stem-like cancer cells to overcome resistance and to improve the clinical outcome of SCLC patients has become a desirable therapeutic approach [59, 60]. However, bioenergetic features and the metabolic adaptation capabilities of CSCs are still not completely understood; they require further investigation in several tumor types, including SCLCs [61, 62].

## Impact of genetic alterations and molecular subtypes on metabolism and response to therapy

As it has been described in several studies, specific genetic alterations can produce distinct metabolic liabilities in cancer. Inactivating mutation of the tumor suppressor TP53 is a near-ubiquitous event [1]; moreover, amplification of *MYC* family members (*MYC*, *MYCL*, and *MYCN*) or genetic alterations resulting in the hyperactivation of mTOR pathway also frequently occur in SCLC cases [27, 63]; and all of these mutations can influence the metabolic phenotype (Fig. 1).

Despite the PI3K/Akt/mTOR pathway is known as a master regulator of cellular metabolism [64], only limited data are available about the associations among mTOR pathway alterations, bioenergetics processes, and newly described molecular subtypes in SCLC. It has been found that SCLC-Y is more sensitive to mTORC1 inhibitor rapamycin [65], probably by the higher incidence of mTOR hyperactivation in this subtype. Cisplatin-resistant SCLC cell lines harboring activating mutations of the mTOR pathway, particularly *PIK3CA* mutation, have been suppressed significantly by *PIK3CA* silencing and the PI3K/mTOR kinase inhibitor NVP-BEZ235, accompanied by the inhibition of Akt and ribosomal protein S6 phosphorylation *in vitro* [30]. SCLC cells with *RICTOR* copy number gain have also shown increased sensitivity to mTOR kinase inhibitors that can suppress the activity of both mTOR kinases [33]. Overexpression and increased activation of the insulin-like growth factor-1 receptor (IGF-1R) has also been observed in SCLC [66] and can result in the activation of the PI3K/Akt/mTOR pathway [67]. IGF-1R has been described as an essential kinase in the SCLC-P subgroup, and IGF-1R inhibitor (linsitinib) treatment has shown significant antiproliferative effect on SCLC cell lines with high *POU2F3* expression (SCLC-P subtype) [68].

Besides the PI3K/Akt/mTOR pathway, *MYC* family alterations can also support biomass production by increasing the metabolic flux through glycolysis, glutaminolysis, TCA cycle, and nucleotide synthesis [26, 69]. *MYC*-driven subset of SCLCs has been recently discovered as highly dependent on arginine biosynthetic pathways suggesting that arginine depletion represent a subtype-specific metabolic vulnerability in these cases. Additionally, chemo-resistant SCLC cells also have exhibited increased *MYC* expression and similar metabolic characteristics to *MYC*-altered chemo-naïve cell lines. Therefore, arginine depletion may represent a promising therapeutic option even in relapsed cases [63]. It has also been described that *MYC* amplification frequently cooperates with *TP53* and *RB1* loss to promote SCLC with aggressive, highly metastatic features, especially in the SCLC-N subtype characterized by high *NEUROD1* expression [70]. Besides arginine, alterations in the *MYC* family members also affect the metabolism of other amino acids, such as glutamine. Dependence on glutaminolysis has been described to be related to *MYC* or *MYCL* amplification; *MYC* have promoted glutamine-dependent lipogenesis, and glutamine withdrawal has been associated with decreased proliferation and increased cell death of *MYC* or *MYCL* overexpressing SCLC cells [71].

Synthesis of purine nucleotides has also been described to be dependent on the expression of *MYC* family members. Elevated guanosine nucleotide level and abundant expression of guanosine biosynthetic pathway proteins (IMPDH1 and 2) have been reported in SCLC cases with low expression of *ASCL1* and high expression of *MYC* [51]. *MYC* have promoted synthesis of guanosine triphosphate (GTP), which is also required for ribosome biogenesis, and thereby protein synthesis. Based on these observations, dependence on inosine monophosphate dehydrogenase, a key enzyme of GTP synthesis, may represent a therapeutic vulnerability in *MYC*-high SCLC cells with acquired chemoresistance [72]. In addition, *MYC* also influences serine and one-carbon metabolism, mainly through the deletion of its obligate heterodimerization partner MAX, which is known as a candidate tumor suppressor and frequently affected in SCLC [73].

*TP53* mutations also can influence cellular metabolism; however, these alterations are not subtype-specific because of the near-ubiquitous nature of *TP53*-loss in SCLC [1]. *TP53* codes for the protein p53 that acts mainly as a transcription factor controlling cell cycle arrest, DNA repair, apoptosis, and senescence [69]. Besides these functions, p53 has been linked to multiple metabolic pathways, such as glycolysis, mitochondrial respiration, lipid and amino acid metabolism, and nucleotide synthesis [74]. The impact of TP53 mutation on cellular metabolism can depend on the subtype of the mutation [75] (e.g., the missense mutation R175H is known to affect mitochondrial biogenesis and oxidative phosphorylation [76, 77]); however, the role of these variants in the regulation of SCLC metabolism is still largely unknown. Moreover, growing evidence supports that p53 and the liver kinase B1 (LKB1)-adenosine monophosphate-activated protein kinase (AMPK)-mTOR pathway are intertwined, thereby p53 also contributes to energy sensing and coordinates bioenergetic processes in a context-dependent manner [74, 78].

## The function of the microenvironment in metabolism and progression of small cell lung carcinoma

**Fig. 2:**
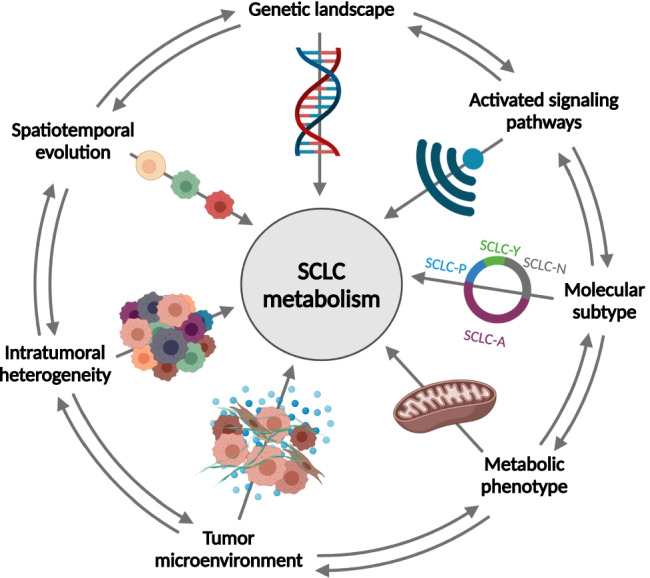
Determinants of SCLC metabolism. The metabolism of SCLC cells is regulated not only by the so-called cell-intrinsic factors, such as genetic background and activated signaling pathways, but also by several other factors that include molecular subtype, metabolic phenotype, tumor microenvironment, intratumoral heterogeneity, and spatiotemporal evolution [79]. Interactions between more or all determinants can be present in the tumor as it is indicated by arrows on the rim of the figure (it cannot be accurately visualized in a 2D figure, but interactions among non-adjacent factors can also be present at the same time)

Recent studies have highlighted that cellular metabolism is regulated not only by cell-intrinsic factors, such as genomic landscape and activated signaling pathways, but also by metabolite availability, heterogeneity, interactions with the surrounding non-cancerous cells, and whole body metabolic homeostasis [79] (Fig. 2). The metabolic niche is composed of cancer cells and a variety of non-cancerous cells in the tumor microenvironment (TME) forming a unique metabolic landscape. Because of the deregulated physiological properties, insufficient blood flow, and the presence of cancer-associated cells (e.g., immune cells), TME is characterized by hypoxic and acidic conditions, nutrient deprivation, electrolyte imbalance, inflammation, and increased oxidative stress [79, 80]. Metabolic crosstalk within the TME may help the adaptation and survival of tumor cells and thereby the progression of the disease.

Despite its emerging role in cancer biology, limited data are available about the TME metabolism in SCLC. Most of the studies focus on immune microenvironment and hypoxia. It has been described that SCLC subtypes with low NE marker expression are characterized by increased immune cell infiltration and elevated expression of the immune checkpoints poliovirus receptor and indoleamine 2,3-dioxygenase, which may represent biomarkers and potential therapeutic targets in SCLC [81]. Recently, a new SCLC subtype with distinct biology and TME (SCLC-I) has been described as characterized by an inflamed gene signature and elevated expression of certain immune genes, particularly those that are related to cytotoxic T lymphocytes [82]. SCLC-I subtype tends to be more responsive to immunotherapy, similarly to SCLC-Y subtype since *YAP1* upregulates PD-L1 expression and induces an immunosuppressive TME [83, 84]. The SCLC-I subtype is also characterized by low expression of ASCL1, NEUROD1, and POU2F3; however, the potential overlap between SCLC-Y and SCLC-I cases has not been described yet [85]. Metabolic reprogramming of cancer cells can also regulate immune response and immune cell fate through the energetic interplay and metabolic competition between cancer and immune cells that limits nutrient availability and causes acidic TME, thereby hinders immune cell functions [86].

Multiple studies underline the presence of hypoxia (or pseudohypoxia) in SCLC cases. An immunohistochemical analysis has revealed that SCLCs also include hypoxic regions [87]. Hypoxia may be caused by dysfunctional vascularization [79] or chemotherapy-induced anemia [88]; furthermore, concomitant chronic obstructive lung disease or even the tumor mass itself can result in airway obstruction and difficulties in blood oxygenation in SCLC patients [89]. An overall lack of hypoxia-inducible factor (HIF)-2α expression has been found in an analysis focusing on the expression of HIF-1α and HIF-2α proteins in SCLC; however, strong expression of HIF-1α has been observed in most cases, particularly adjacent to necrotic tumor regions. Similarly, accumulation of HIF-1α and no or low level of HIF-2α have been found at hypoxia in SCLC cell lines; moreover, SCLC cells were able to survive even modest and severe hypoxia *in vitro* [90]. Another study has found HIF-1α and HIF-2α expression in 48.9% and 24.4% of the SCLC patients, respectively. HIF-2α expression has been associated with necrotic regions, whereas HIF-1α expression has been diffusely distributed within the tumor nests. HIF-2α expression has also been correlated with tumor growth and the presence of distant metastasis; moreover, both HIF-1α and HIF-2α expressions have been related to decreased overall survival [91].

Metabolism of hypoxic tumors mostly relies on glycolysis [92]; therefore, targeting lactate transport by inhibiting of monocarboxylate transporters (MCTs) seems to be an attractive strategy for the treatment of these tumors. The expressions of MCT1 and the hypoxia marker carbonic anhydrase IX were found in the absence of MCT4 expression in 21% of the SCLC cases. Additionally, it was demonstrated that MCT1 inhibitor (AZD3965) has a significant inhibitory effect on tumor growth and increased intratumoral lactate level in hypoxic SCLC cells lacking expression of the alternative lactate transporter MCT4 [93].

## Contribution of mTOR pathway and bioenergetic alterations to temporal evolution, therapy resistance, and progression of small cell lung carcinoma

Intratumoral metabolic heterogeneity and plasticity are cornerstones of tumor evolution, therapy resistance, and metastasis formation [94, 95]. The metastatic cascade, including both epithelial-to-mesenchymal transition (EMT) and colonization of different metastatic sites, also represents metabolic challenges for the disseminating tumor cells [96].

**Fig. 3:**
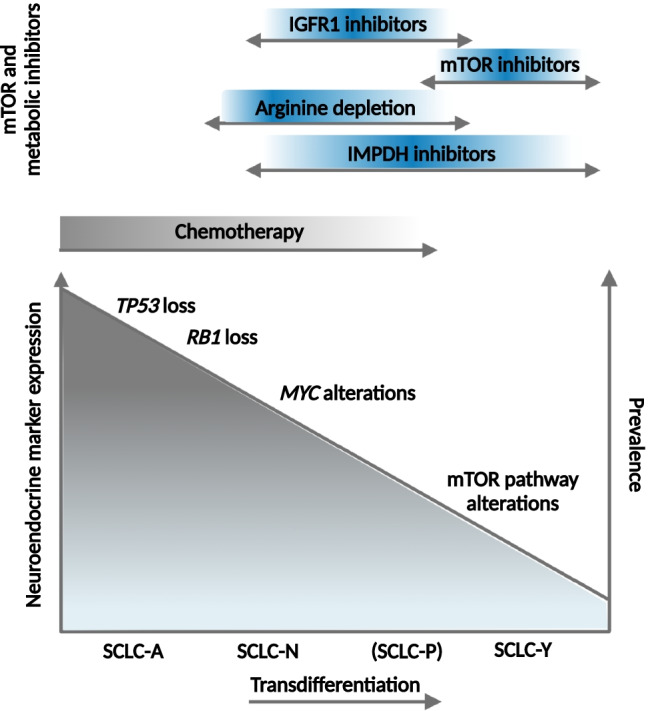
Therapeutic targets and treatment opportunities during the transdifferentiation of SCLC. A recent study has described that there is a temporal shift from SCLC-A to SCLC-N then to SCLC-Y subtypes instead of a permanent subtype [97]. This transdifferentiation process inversely correlates with the prevalence of the cases as well as the NE-marker expression. Each step (or subtype) is characterized by distinct vulnerabilities that can be therapeutically exploited. Acquired resistance to platinum-based chemotherapy (marked with gray color) develops in almost all cases; however, mTOR and metabolic alterations may provide additional therapeutic targets in resistant SCLC cases (marked with blue color)

SCLC is one of the tumor types with the highest metastatic proclivity that determines the high mortality of the disease. SCLC tumor cells can arise from different cell types of origin with distinct genetic and epigenetic characteristics that underlines the presence of intertumoral heterogeneity and subtype-specific differences in tumor evolution and metastatic progression [9]. Distinct SCLC subtypes have been defined based on the expression of the transcription factors *ASCL1*, *NEUROD1*, *POU2F3*, and *YAP1* [6]; however, a recent study has revealed that instead of a permanent subtype, there is a *MYC*-driven temporal shift from *ASCL1*-positive to *NEUROD1*-positive and then to *YAP1*-positive states (Fig. 3). Moreover, SCLCs have been found to be composed of multiple molecular subtypes supporting that dynamic temporal evolution can also occur *in vivo* [97]. Another study analyzing circulating tumor cell-derived xenografts by single-cell RNA sequencing has also found increased intratumoral heterogeneity after treatment resistance [8]. These findings emphasize the importance of intratumoral heterogeneity in cancer progression and treatment resistance as well as the need for treatment strategies that target heterogeneity and the divergent evolvement of tumor cells, thereby may counteract spatiotemporal evolution, and therapy resistance.

Metastatic ability of SCLC is regulated by several factors, including cell type of origin, genetic and epigenetic landscape, TME, cell fate, and inter- and intratumoral heterogeneity [10]. Comprehensive bioinformatic analyses have shown that adherent and semiadherent, chemoresistant SCLC cells are enriched with alterations affecting the PI3K/Akt/mTOR pathway; furthermore, activated PI3K/Akt/mTOR pathway has promoted the phenotypic transition from suspension to adhesion growth pattern as well as the development of chemoresistance. PI3K/mTOR inhibitors combined with chemotherapy have synergistically inhibited the growth of SCLC cells, which underlines the role of this combination in relapsed cases [98]. The importance of the mTOR pathway, particularly mTORC2, in the progression and metastasis formation has also been highlighted in our immunohistochemical analysis, in which high expression of both Rictor (the scaffold protein of mTORC2) and phospho-(Ser473)-Akt (the downstream target of mTORC2) has been associated with unfavorable clinical outcome in SCLC patients. In correlation with its negative impact on overall survival, high expression of Rictor has also been associated with metastatic disease [31]. In line with these observations, the higher migration ability of SCLC cells with *RICTOR* copy number gain has also been reported *in vitro* [33]. Also, *YAP1* expression, the characteristic marker of SCLC-Y subtype, has already been described to promote proliferation, invasion, and metastasis in NSCLC cell lines [99]. Since SCLC-Y is more sensitive to mTOR inhibitors [65], it is conceivable that there is a significant overlap between *YAP1*-positive, chemoresistant, highly metastatic cases, and those enriched with mTOR pathway alterations.

There is only limited data available about the associations of treatment resistance, metastasis formation, and metabolic processes. Cisplatin treatment has resulted in metabolic rewiring and distinct metabolic profile in resistant SCLC cell populations; moreover, it has also been described that hypoxia and enhanced glycolysis may contribute to the development of cisplatin resistance and recalcitrant behavior of SCLC [100].

## Contribution of mTOR pathway and site-specific metabolic alterations to the metastatic potential of small cell lung carcinoma

Site-specific metabolic features suggest that tumors that arise in different organs have to adapt to diverse microenvironments and might have distinct metabolic dependencies and vulnerabilities, as well [79]. This might explain the “seed and soil” hypothesis of metastasis formation, the unique preference of cancer cells to certain metastatic sites with distinct genetic, epigenetic, physiological, and metabolic conditions.

Analyzing lung adenocarcinomas, it has been described recently that high-plasticity cell state drives cellular heterogeneity, which arises independently of genetic alterations [101]. Cellular plasticity also plays an important role in tumor progression and metastasis formation; in addition, it implies the ability of the cancer cells to adapt to microenvironmental changes [102]. In contrast, it has also been reported that tumor cells can induce changes in the TME by tumor-secreted factors and extracellular vesicles before their arrival that can help their adaptation and survival [103]. This so-called pre-metastatic niche has characteristic metabolic changes that can contribute to organotropism, as well. It has been recently described that while circulating tumor cells mostly depend on enhanced antioxidant metabolism, the pre-metastatic niche can rely on glucose availability [104]. There is no specific information available on the role of pre-metastatic niche in SCLC. However, studies have also emphasized the role of lipid metabolism in metastatic progression of various tumor types with similarly high metastatic capability such as melanoma and ovarian cancer [105–107].

SCLCs frequently metastasize to the brain, liver, bone, and lymph nodes; however, little is known about the cellular and molecular mechanisms and preferences that drive the colonization of the specific metastatic organ microenvironments [10]. Most of the available data are about brain metastases that represent one of the major causes of SCLC mortality. Brain metastases are present in 15–20% of the SCLC patients at the time of the diagnosis [108]; furthermore, they will develop in an additional 40–50% of the patients during the course of the disease [108]. High availability of glucose in the central nervous system may help the growth and proliferation of highly glycolytic cancer cells in the brain [109]. SCLC is characterized by high glucose uptake [40, 43] that may contribute to the high frequency of brain metastases in SCLC patients. Despite the growing evidence of the interplay between metabolic rewiring and the metastatic process, there is no specific information about the role of metabolic plasticity and bioenergetic processes in the metastasis formation of SCLC, but a few studies are available about the role of the mTOR pathway in the metastatic cascade.

The PI3K/Akt/mTOR pathway is frequently affected in SCLC [28, 30] and may also contribute to the metastatic progression of the disease, including the development of brain metastases. We have already described that mTOR activity is higher in brain metastases of lung adenocarcinomas as compared to primary tumors. Additionally, high expression of Rictor, the characteristic scaffold protein of mTORC2, has predicted subsequent metastasis formation [110]. A recent study analyzing matched primary and metastatic lung cancer pairs has revealed that *RICTOR* amplification is associated with metastatic tumors [111], implying the potential of this genetic alteration to drive the metastatic process. Similarly, we found higher Rictor expression in distant metastases as compared to lymph node metastases and primary tumors in a study analyzing 100 SCLC cases [31]. Activation of the PI3K/Akt/mTOR pathway has been described to play an important role in early metastatic colonization of the brain in several tumor types, including SCLC [112]. Based on these data, it is conceivable that inhibition of the mTOR pathway may prevent or decrease metastasis formation in SCLC patients.

## mTOR pathway and metabolic inhibitors as a new therapeutic approach in the management of small cell lung carcinoma

Despite the promising *in vitro* results listed above have predicted the efficacy of mTOR inhibitors in SCLC, early clinical studies have demonstrated limited antitumor properties. RADIANT and LUNA studies have found that the mTORC1 inhibitor everolimus has a clinically meaningful antitumor activity and increases the progression-free survival in low- or intermediate-grade lung neuroendocrine tumors even in monotherapy or in combination with pasireotide [113–115]. In SCLCs, however, mTORC1 inhibitors (both everolimus and temsirolimus) have shown limited antitumor activity as single agents in unselected patient populations [116, 117]. Patients are currently recruited for a study to assess the efficacy of sirolimus combined with the antirheumatic agent auranofin (Table 2).

**Table 2:** Ongoing, completed and terminated clinical trials with mTOR and metabolic inhibitors in small cell lung carcinoma

	Target	Drug	In combination with	Cancer types	Line	Phase	Status or result	ClinicalTrials.gov identifier	References
mTOR pathway inhibitors	mTORC1	RAD001 (everolimus)	Cisplatin, etoposide	Lung cancer	First	I	Completed (feasible everolimus dose: 2.5 mg/kg; best overall response: partial response)	NCT00466466	[118]
	mTORC1	RAD001 (everolimus)	Carboplatin, etoposide	Small cell lung cancer, other advanced solid tumors	First/second	I	Terminated (reason: number of known toxicities observed despite a treatment-naïve population)	NCT00807755	
	mTORC1	RAD001 (everolimus)		Small cell lung cancer (previously treated)	Second/third	II	Completed (well tolerated, but limited single agent efficacy)	NCT00374140	[117]
	mTORC1	Sirolimus	Auranofin	Non-small cell lung cancer or small cell lung cancer (advanced or recurrent)	Second	I/II	Recruiting	NCT01737502	
	mTORC1	Temsirolimus		Small cell lung cancer (extensive-stage)	Second	II	Completed (no increase in progression-free survival	NCT00028028	[116]
	mTORC1	Temsirolimus	Vinorelbine ditartrate	Unresectable or metastatic solid tumors	First/second	I	Completed (no results available)	NCT01155258	
	mTORC1/C2	Vistusertib		Small cell lung cancer (relapsed, harboring RICTOR Amplification)	Second	II	Terminated (reason: decision of IP support organization)	NCT03106155	
	mTORC1/C2	Vistusertib	Navitoclax	Small cell lung cancer (relapsed), other solid tumors	First/second	I/II	Active, not recruiting	NCT03366103	
	PI3K	BKM120	Cisplatin, etoposide	Small cell lung cancer, other advanced solid tumors	First/second	I	Completed (no results available)	NCT02194049	
	Akt	MK-2206		Non-small cell lung cancer, small cell lung cancer, thymic malignancies	First/second	II	Active, not recruiting	NCT01306045	
Metabolic inhibitors	Arginase 1 deficiency	ADI-PEG 20		Small cell lung cancer (relapsed sensitive or refractory)	Second/third	II	Terminated (reason: lack of efficacy in Cohort 2; slow enrollment in Cohort 1)	NCT01266018	
	Arginase 1 deficiency	ADI-PEG 20	Gemcitabine, docetaxel	Soft tissue sarcoma, osteosarcoma, Ewing's sarcoma, small cell lung cancer	Second	II	Active, not recruiting	NCT03449901	
	Arginase 1 deficiency	Pegzilarginase	Pembrolizumab	Small cell lung cancer (extensive-stage)	Second	I/II	Completed (no results available)	NCT03371979	
	IGF-1R	Figitumumab	Cisplatin/carboplatin, etoposide	Small cell lung cancer (extensive-stage)	Second	II	Terminated (reason: low participants enrollment and the halting of the figitumumab development program)	NCT00977561	
	IGF-1R	Linsitinib		Small cell lung cancer (relapsed)	Second	II	Completed (safe, but no clinical activity in unselected patients)	NCT01533181	[119]
	TCA-cycle	CPI-613 (6,8-bis(benzylthio)octanoic acid)		Small cell lung cancer (relapsed or refractory)	Second	I	Completed (no efficacy as a single agent)	NCT01931787	[120]
	AMPK, oxygen consumption	Metformin	Sintilimab	Small cell lung cancer	Second	II	Recruiting	NCT03994744	
	HMG-CoA reductase	Simvastatin	Cisplatin, irinotecan	Small cell lung cancer (extensive-stage)	First	II	Completed (no increase in survival, possible efficacy in heavy smokers)	NCT00452634	[121]
	HMG-CoA reductase	Simvastatin	Cisplatin, irinotecan	Small cell lung cancer (extensive-stage, chemo-naïve patients)	First	II	Recruiting	NCT01441349	
	HMG-CoA reductase	Simvastatin	Irinotecan	Small cell lung cancer (extensive-stage, relapsed)	Second	II	Not Yet Recruiting	NCT04985201	
	HMG-CoA reductase	Simvastatin	Albumin paclitaxel	Small cell lung cancer (extensive-stage, relapsed)	Second	II	Recruiting	NCT04698941	
	HMG-CoA reductase	Pravastatin	Etoposide, cisplatin/carboplatin	Small cell lung cancer	First	II	Completed (safe, but no survival benefit)	NCT00433498	[122]

*Akt* protein kinase B, *HMG-CoA* 3-hydroxy-3-methyl-glutaryl-coenzyme A, *IGF-1R* insulin-like growth factor 1 receptor, *mTORC1* mammalian target of rapamycin complex 1, *mTORC1/2* mammalian target of rapamycin complex 1/2, *PI3K* phosphatidylinositol 3-kinase, *TCA* tricarboxylic acid

Next-generation inhibitors of the mTOR kinase target both mTOR complexes (mTORC1 and mTORC2) resulting in reduced activity of the mTOR pathway and, in contrast to rapalogs, have the potential to avoid the feedback loop-based activation of Akt [123]. An umbrella study (SUKSES-D) has started to study the efficacy of the vistusertib (mTORC1/C2 inhibitor) in *RICTOR*-amplified SCLC; however, after enrolling 4 patients, the study has been terminated without significant results [124]. Vistusertib is still under investigation in combination with navitoclax (Blc-2 inhibitor) in patients with relapsed SCLC (Table 2). Onatasertib (CC-223), another mTORC1/2 inhibitor, has shown encouraging results in metastatic non-pancreatic gastrointestinal NE tumors [125]; but it has not been tested yet in NE tumors of the lung. Sapanisertib (INK-128) and AZD8055 are mTORC1/2 inhibitors with promising results in advanced solid tumors; unfortunately, no study is available with these drugs focusing on SCLCs. JR-AB2-011 is a selective inhibitor of mTORC2 that blocks the interaction of Rictor and mTOR, thereby the assembly of mTORC2 [126]. Beside it has demonstrated significant antitumor properties in preclinical models of glioblastoma [126], JR-AB2-011 has also reduced melanoma cell migration and metastasis formation in an *in vitro* and *in vivo* study [127]. These results suggest that inhibition of Rictor-mTOR interaction may represent a promising therapeutic approach in mTORC2-hyperactivated, highly metastatic tumors, such as *RICTOR*-amplified SCLCs.

Other inhibitors of mTOR pathway (e.g., PI3K and dual PI3K/mTOR inhibitors) are also under investigation in SCLC, mostly in preclinical phase [128, 129]. The pan-class I PI3K inhibitor buparlisib has been tested in SCLC in combination with cisplatin and etoposide in a phase I clinical study; however, no study results are available (NCT02194049). An ongoing phase II study is investigating the Akt inhibitor MK-2206 in *PIK3CA*, *PTEN*, and *AKT*-mutant thoracic malignancies including SCLCs (NCT01306045) that are hypothesized to exhibit mTOR pathway hyperactivation, thereby may have an increased sensitivity to the drug.

SCLCs are associated with characteristic bioenergetic alterations that may also represent therapeutic vulnerabilities and can be exploited in the treatment of SCLCs. One of these metabolic liabilities is arginine dependence, which is associated with the lack of ASS1 expression in a subset of SCLC patients [56]. Besides the mTOR pathway, increased reliance on the essential amino acid arginine may also contribute to cisplatin resistance [63]. After encouraging preclinical results, the efficacy of pegzilarginase, a recombinant human arginase 1 enzyme, has been tested in combination with the PD-1 inhibitor pembrolizumab in SCLC patients, but no results have been released (NCT03371979). Another drug that selectively targets ASS1-deficient cancer cells is ADI-PEG20, which is an arginine deiminase (ADI) PEGylated with 20,000 molecular weight polyethylene glycol (PEG) [130]. A phase II clinical trial using ADI-PEG20 monotherapy in ASS1-deficient SCLC has failed to meet its primary endpoint [54]. An ongoing phase I/II study is investigating the efficacy of ADI-PEG20 in combination with gemcitabine and docetaxel in SCLC patients (NCT03449901).

IGF-1R inhibitors may also represent a relevant therapeutic opportunity in SCLC. Preclinical studies have shown promising results with IGF-1R inhibitors in SCLC [131, 132]; however, a phase II clinical trial has failed to prove efficacy of the IGF-1R inhibitor linsitinib (OSI-906) in unselected SCLC patients [119]. The importance of IGF-1R has been described in the SCLC-P subtype; therefore, biomarker-driven patient selection may improve clinical outcomes [68]. Figitumumab, a human monoclonal antibody against IGF-1R, has been studied in combination with etoposide and cisplatin (or carboplatin). Although safety concerns have not been seen, the phase II trial has been discontinued due to slow enrollment (NCT00977561).

Dependence on GTP synthesis may represent a new metabolic liability in chemotherapy-resistant SCLC patients, but only preclinical data are available about the efficacy of the IMPDH inhibitor mizoribine in SCLC [51, 72]. Another novel metabolic inhibitor, CPI-613, that target mitochondrial metabolism has not demonstrated single-agent efficacy in SCLC patients in a phase II clinical trial; however, preliminary findings suggest that topotecan may increase antitumor properties of the drug [120].

Drug repurposing is the process of investigation of existing, approved drugs for new therapeutic purposes [133]. Studies are ongoing to assess the repurposing possibilities of the antidiabetic drug metformin and the cholesterol-lowering statins in the treatment of SCLC (Table 2). Metformin use has improved both the overall and the disease-free survival of patients suffering from combined SCLC [134]. Additionally, a recent case report suggests that metformin may also have the ability to overcome acquired resistance to PD-1 inhibitors in SCLC [135]. A phase II clinical study is recruiting patients to assess the safety and efficacy of metformin combined with the PD-1 inhibitor sintilimab in SCLC (NCT03994744). Preclinical results with simvastatin have also shown significant effects on SCLC cell growth and survival [136]. In contrast, the LUNGSTAR phase III clinical trial adding pravastatin to first-line standard chemotherapy has not confirmed these promising results [122]. A few studies are still recruiting patients to study the efficacy of simvastatin combined with cisplatin, irinotecan, or paclitaxel (Table 2).

## Concluding remarks and future directions

Although SCLC initially responds to the first-line platinum-based chemotherapy, acquired resistance develops in almost all patients determining the recalcitrant behavior of the disease [137]. During the last 2 decades, we have witnessed a paradigm shift in the treatment of NSCLCs, and despite the initial nihilistic attitude, the pace of SCLC research has also been accelerated in the past few years. However, limited availability of fresh SCLC tissue is still a significant barrier for translational research [138]. Surgical resection is rarely performed as a part of therapy, and, therefore, most of the studies can only rely on archival tissue, small-biopsy samples, or explant models such as patient-derived xenografts and cell lines, which may have limited applicability to the biology of the original tumor. Similarly, relatively few murine models of SCLC exist, and they do not always fully recapitulate human disease. These barriers have contributed to difficulties in both understanding the disease and translating knowledge to effective therapies. Still, recent advances in understanding SCLC biology in the past years have led to the discoveries of characteristic genetic alterations, activated signaling pathways, and molecular subtypes of SCLC, which may serve as therapeutic targets in personalized therapy [1].

Recently, the mTOR kinase has been identified as an essential kinase in SCLC [36], and the therapeutic priority of mTOR pathway inhibitors has been suspected based on the high frequency of genetic alterations affecting the PI3K/Akt/mTOR pathway in SCLC [30], which are hypothesized to result in mTOR hyperactivation in cancer cells. *RICTOR* amplification has been identified as the most common targetable genetic alteration in SCLC cases [27, 31, 33], and its possible role in metastasis formation [111, 127] underlines the need for biomarker-driven clinical studies with mTORC1/2 or even selective mTORC2 inhibitors in case the latter will also be available for clinical use.

Targetable metabolic vulnerabilities of SCLC have also been revealed in the past decade. Since the mTOR pathway is known as a master regulator of cellular metabolism [18], most of these bioenergetic liabilities are interconnected with the PI3K/Akt/mTOR pathway. One of these promising targets is the arginine dependence in a subset of SCLCs, which is related to the ASS1-deficiency of the cancer cells and can be therapeutically exploited [54]. The intracellular level of certain amino acids, such as arginine and leucine, regulates the activity of mTORC1; therefore, disturbances in arginine biosynthesis may also influence the mTOR pathway [139, 140].

Both mTOR and metabolic inhibitors have been investigated in clinical trials; however, to date, most of them showed limited single-agent efficacy. Early clinical data suggest that the development of rational drug combinations and biomarker-based patient selection may improve efficacy and help to avoid treatment resistance [27]. It has been found that SCLC subtypes have distinct therapeutic vulnerabilities (Fig. 3) [141], but recent studies have described that instead of a permanent subtype, there is a transdifferentiation from SCLC-A to -N- then to -Y subtype that may also influence therapeutic response. Additionally, intratumoral heterogeneity and the presence of multiple subtypes and vulnerabilities within the same tumor may also represent limitations of the therapy and can also contribute to resistance and progression of the disease [97].

In summary, recent advances in the understanding of SCLC biology are encouraging, and, after a gap of many years, these have led to new therapeutic opportunities in the treatment of the disease. Unfortunately, most of these therapeutic options have shown limited efficacy in monotherapy, which draw attention to the development of rational combination strategies. Intratumoral heterogeneity and spatiotemporal evolution of the cancer cells can lead to acquired resistance, progression, and metastasis formation; therefore, these have to be considered in future therapeutic approaches.
